# Single and combined effects of cisplatin and doxorubicin on the human and mouse ovary *in vitro*

**DOI:** 10.1530/REP-19-0279

**Published:** 2019-12-09

**Authors:** Federica Lopes, Jin Liu, Stephanie Morgan, Rebecca Matthews, Lucy Nevin, Richard A Anderson, Norah Spears

**Affiliations:** 1 Biomedical Sciences, University of Edinburgh, Edinburgh, UK; 2 Department of Public Health, Fujian Medical University, Fuzhou, China; 3 MRC Centre for Reproductive Health, University of Edinburgh, Edinburgh, UK

## Abstract

Chemotherapy drugs are administered to patients using combination regimens, and as such the possibility of multiplicative effects between drugs need to be investigated. This study examines the individual and combined effects of the chemotherapy drugs cisplatin and doxorubicin on the human ovary. Although cisplatin and doxorubicin are known to affect female fertility, there is limited information about their direct effects on the human ovary, and none examining the possibility of combined, multiplicative effects of co-exposure to these drugs. Here, human ovarian biopsies were obtained from 14 women at the time of caesarean section, with 38 mouse ovaries also obtained from neonatal C57Bl/6J mice. Tissue was cultured for 6 days prior to analyses, with chemotherapy drugs added to culture medium on the second day of culture only. Treatment groups of a single (5 μg/mL human; 0.5 μg/mL mouse) or double (10 μg/mL human; 1.0 μg/mL mouse) dose of cisplatin, a single (1 μg/mL human; 0.05 μg/mL mouse) or double (2 μg/mL human; 0.1 μg/mL mouse) dose of doxorubicin or a combination of a single dose of both drugs together were compared to controls without drug exposure. Exposure to cisplatin or doxorubicin significantly decreased follicle health in human and mouse, supporting the suitability of the mouse as a model for the human ovary. There was also a significant reduction of mouse follicle number. Human ovarian stromal tissue exhibited increased apoptosis and decreased cell proliferation. Crucially, there was no evidence indicating the occurrence of multiplicative effects between cisplatin and doxorubicin.

## Introduction

Women who have been treated with chemotherapy drugs for cancer have an increased chance of experiencing fertility problems after treatment (Letourneau *et al*. 2012, Barton *et al*. 2013, Chow *et al*. 2016, van Dorp *et al*. 2018), and a reduced chance of pregnancy (Anderson *et al*. 2018). Given the improving survival rates for most cancers, including childhood cancers and those affecting younger women, chemotherapy-induced infertility is affecting an increasing number of people in our population. Despite this, relatively little information is available about the effects of chemotherapy drugs on the human ovary, with most of our understanding based on animal studies or extrapolated from clinical outcomes, themselves often using indirect indices of fertility such as return of menses after treatment. Chemotherapy treatment has been shown to affect follicle health and developmental capacity (Asadi Azarbaijani *et al*. 2015, Pampanini *et al*. 2019). Where information about effects of experimental exposure to chemotherapeutics on the human ovary is available, it details the effect of exposure to a single chemotherapy drug at a time, whereas cancer treatment generally involves combination regimens. Treatment with multiple drugs is an effective strategy to reduce drug resistance and side effects. However, the combined effects of exposure to multiple drugs is not always predictable, with the possibility of additive or even more complex multiplicative effects, a chance that can be exacerbated by a large number of potential drug/dose combinations (Tallarida *et al*. 1989).

Cisplatin (CIS) and doxorubicin (DOX) are two commonly co-administered chemotherapy drugs, used in the treatment of a wide range of cancers including breast (Di *et al*. 2016), bladder (Chen *et al*. 2012), endometrial, lung and ovarian (Tas *et al*. 2008) cancers, as well as in lymphoma, neuroblastoma, sarcoma and a range of paediatric cancers (Pritchard *et al*. 2000, Kobys *et al*. 2013).

There is clear evidence that ovarian exposure to either CIS or DOX results in dose-dependent follicle loss and an increase in apoptosis, but the vast majority of our knowledge come from animal model studies, primarily using a mouse model. Immature oocytes are particularly susceptible to CIS exposure, resulting in follicular atresia (Morgan *et al*. 2013, Tuppi *et al*. 2018). The store of resting, primordial follicles is reduced after exposure, with evidence both of a direct damaging effect of CIS on primordial follicles (Nguyen *et al*. 2018, 2019), and of an indirect effect on primordial follicles resulting from accelerated activation of primordial follicles due to a loss of inhibitory factors previously secreted by more developed follicles that have themselves been targeted by the drugs (‘burnout’ (Kalich-Philosoph *et al*. 2013)); currently, there is some controversy over the extent to which each of these different pathways is primarily responsible for the loss of primordial follicles. DOX exposure results in a loss of both primordial and growing follicles, mainly through effects on mitotically active granulosa cells (GCs), and leads to reduced ovulation rates (Perez *et al*. 1997, Ben-Aharon *et al*. 2010, Morgan *et al*. 2013, Roti Roti *et al*. 2014). DOX also damages ovarian microvasculature and induces necrosis of stroma cells (Ben-Aharon *et al*. 2010). Direct information about the effects of CIS or DOX on the human ovary is more limited, but data are available using culture or xenotransplantation of human cortical strips and also culture of ovarian cells or cell lines. Exposure to either CIS or DOX leads to a loss of follicles, including ones at the resting primordial stage, with research using cultured and xenotransplanted ovarian biopsies exposed to CIS (Bildik *et al*. 2015, 2018) and xenotransplantation after treatment with DOX (Soleimani *et al*. 2011, Li *et al*. 2014). CIS exposure increases apoptosis of human ovarian GCs, as has been shown in work using either primary cultures of luteinised GCs (Chatterjee *et al*. 2014, Bildik *et al*. 2018) or GC lines (Yuksel *et al*. 2015), with Bildik *et al*. (2018) also showing a reduction in luteinised GC proliferation rates. There are thus no data relating to normal, follicular GCs. In addition, CIS and DOX both reduce steroidogenic activity (CIS (Chatterjee *et al*. 2014, Bildik *et al*. 2015, Yuksel *et al*. 2015) and DOX (Imai *et al*. 2007)).

Less is known about the effect of chemotherapy drugs on the stromal compartment of the human ovary, despite the fact that damage to stromal tissue, particularly to the vasculature, can have a downstream effect on follicle health and development (Meirow *et al*. 2007). CIS and DOX treatments both damage the ovarian stromal vasculature (CIS (Bildik *et al*. 2015); DOX (Soleimani *et al*. 2011)). Only one study has examined the effects of both CIS and DOX in the same system, although that research examines the effects of each drug separately, with either CIS or DOX inducing apoptosis in cultured primary stromal cells (Fabbri *et al*. 2016).

Here, we examine the effect of CIS and DOX on the human ovary through culture of ovarian cortical strips, examining the effect of the drugs on both the follicular and stromal compartments. Since CIS and DOX are often co-administered in treatment, in addition to investigating the individual effect of the two drugs, we also examine whether there are any additive or more complex multiplicative effects when the ovary is exposed to both drugs at the same time. To the best of the authors’ knowledge, this study is the first to date that has examined the combined effects of CIS and DOX on both the human ovarian follicular and stromal compartments. With the majority of our current understanding about the effect of the drugs gained from studies using a mouse model and with the limitations inherent in using human samples, we also use the same experimental design to investigate effects of CIS and/or DOX exposure on cultured mouse ovary, to enable comparison of the two systems.

## Materials and methods

### Human ovary tissue

Ovarian cortical tissue samples were collected from 14 healthy women aged 27–34 who were undergoing elective caesarean section, with written informed consent and approval from the Lothian Research Ethics Committee (LREC 10/S1101/2). Ovarian tissue was transported to the laboratory in pre-warmed Leibovitz L-15 medium (Invitrogen) supplemented with sodium pyruvate (2 mM), glutamine (2 mM) (both Invitrogen), HSA (3 mg/mL) (Sigma-Aldrich Ltd), 100 U/mL penicillin and 100 µg/mL streptomycin (P/S; Invitrogen).

### Animals

All work was approved by the University of Edinburgh’s Local Ethical Review Committee and carried out in accordance with UK Home Office regulations. C57Bl/6J mice were housed in a 14 h light:10 h dark photoperiod, with access to food and water* ad libitum*.

### Human ovary tissue culture

Strips of human ovarian cortex were cultured using a method similar to Telfer *et al*. (2008). In order to obtain small and consistently sized fragments of human ovarian tissue for culture, a tissue chopper was used to cut 0.5 mm thick sections of the ovarian biopsy, with a scalpel blade then used to cut 1 × 1 mm fragments from the sections: this work was all carried out in dissecting medium Leibovitz L-15 medium with 3 mg/mL human serum albumin (HSA, Sigma-Aldrich Ltd), 100 U/mL penicillin and 100 µg/mL streptomycin (P/S; Invitrogen), 2 mM L-glutamine (Gibco), 2 mM sodium pyruvate (Sigma-Aldrich Ltd), 2 mM sodium pyruvate (Sigma-Aldrich Ltd). From each ovarian biopsy, at least four ovarian fragments were collected and fixed prior to culture. From the remaining fragments, each piece was then transferred onto Whatman Nucleopore membrane (Camlab Ltd) floating on 1 mL of medium in a 24-well plate, with the thick (1 × 1 mm) side of the fragment placed on to the membrane in order to maximise exposure of the tissue to the gas-liquid interphase, and hence, improve tissue viability. Culture was in McCoy’s culture medium (Invitrogen) supplemented with 1 mg/mL HSA, P/S, 2 mM L-glutamine (Gibco), 5.5 µg/mL transferrin, 5 ng/mL sodium selenite, 10 µg/mL human insulin (ITS, Sigma-Aldrich Ltd), 50 µg/mL ascorbic acid (Sigma-Aldrich Ltd), 0.005 IU/mL FSH (Merk Serono, S.p.A.), and incubated under a controlled atmosphere with 5% CO_2_ at 37°C for 24 h (Day 1). On Day 2, treatment groups were exposed to chemotherapy drug for 24 h only, with CIS (Sigma-Aldrich Ltd) or DOX (Sigma-Aldrich Ltd) added to culture medium as follows: a single dose of one drug (Single CIS – 5 μg/mL; Single DOX – 1 μg/mL), a double dose of one drug (Double CIS – 10 μg/mL; Double DOX – 2 μg/mL), or a combination of a single dose of both drugs together (Combination CIS + DOX – 5 μg/mL CIS plus 1 μg/mL DOX). From each biopsy, four-to-six pieces of tissue were exposed to each treatment, with tissue randomly distributed across the treatments. For each analysis of every experimental treatment, all technical replicates from one patient were combined to give a single datapoint. Tissues were exposed to chemotherapy drugs for a short time period, as in previous work by us and others, to mimic the short period of exposure that patients usually experience each drug cycle (Morgan *et al*. 2013, Lopes *et al*. 2014, 2016, Lande *et al*. 2017, Rossi *et al*. 2017, Smart *et al*. 2018). On Day 3 of culture, after 24 h of drug exposure, tissue from experimental treatments was moved to drug-free medium for 96 h, with medium changed after 48 h: Control tissue was kept in drug-free medium throughout, with medium changed at the same time as for experimental groups. For all groups, and with medium for all experimental group changed on alternative days. For all groups, bromodeoxyuridine (BrdU; Sigma-Aldrich Ltd; at a concentration of 15 µg/mL) was added to the medium for the final 24 h of culture for subsequent determination of cell proliferation. On Day 6 tissues were processed for analyses as detailed below. Cultures were maintained for 4 days following the day of drug exposure to enable histological investigation of the downstream effects of drug exposure on follicle health.

### Mouse ovary tissue culture

Mouse ovaries were cultured as in Lopes *et al*. (2016). Ovaries were obtained from C57Bl/6J mice at postnatal day 4 and dissected into Leibovitz L-15 dissection medium (Invitrogen) supplemented with 3 mg/mL BSA (Sigma-Aldrich Ltd). In total, 38 mouse ovaries were cultured, across four culture runs, with five to eight ovaries analysed per treatment group as detailed below. Each ovary was transferred into 24-well plates onto membranes, as above, floating on 1 mL of α-MEM medium (Invitrogen) supplemented with 3 µg/mL BSA and incubated under a controlled atmosphere with 5% CO_2_ at 37°C for 24 h (Day 1). On Day 2, CIS or DOX was added as follows: a single dose of one drug (Single CIS – 0.5 μg/mL, *n* = 6 Single DOX – 0.05 μg/mL, *n* = 6), a double dose of one drug (Double CIS – 1 μg/mL, *n* = 6; Double DOX – 0.1 μg/mL, *n* = 5), or a combination of single doses of both drugs together (Combination CIS + DOX – 0.5 μg/mL CIS plus 0.05 μg/mL DOX, *n* = 8), with Control ovaries left in drug-free medium (*n* = 7). In the mouse model, lower drug concentrations were required to induce a similar dose response effect to the human tissue, probably due to the less compact nature of the mouse ovarian stroma. After 24 h of drug exposure, ovaries were moved to drug-free medium (Day 3), with Control tissue kept in drug-free medium throughout, and with medium changed on alternative days until Day 6, when tissues were processed for analyses as detailed below. Cultures were maintained for 4 days following the day of drug exposure to enable histological investigation of the downstream effects of drug exposure on follicle health.

### Histology

Paraffin wax blocks were sectioned at 5 µm, and either taken for immunohistochemistry (IHC; details below) following fixation of tissue in Neutral Buffered Formalin (Sigma Aldrich), or photomicrographs taken of haematoxylin and eosin-stained sections (DMLB Leica microscope, Leica Microsystems Ltd) following fixation of tissue in Bouin’s solution (Sigma Aldrich), with stained sections used to undertake morphological examination of ovarian follicles.

### Ovary analysis: follicle counts, stages and health

**Figure 1:**
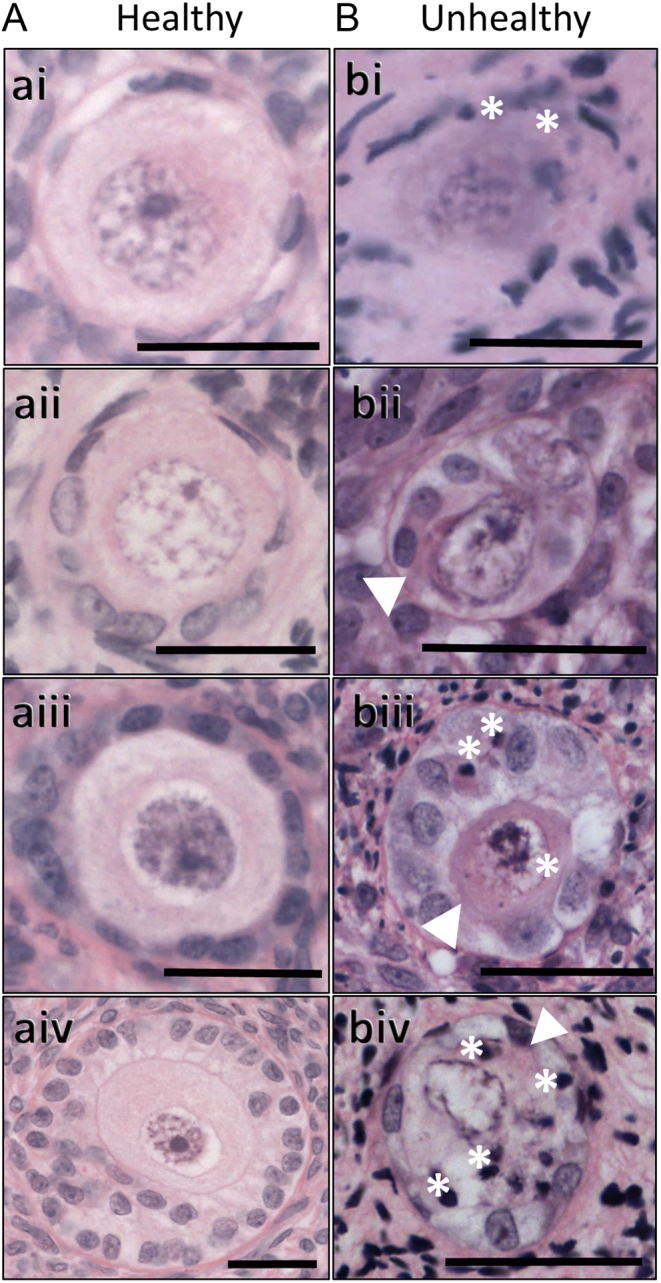
Follicle classification: representative examples of healthy and unhealthy human ovarian follicles at different developmental stages. Photomicrographs of histological sections of follicles from cultured ovaries to show (A) healthy and (B) unhealthy follicles, at the (i) primordial; (ii) transitional; (iii) primary or (iv) secondary stage of development. White asterisks indicate condensed chromatin; white arrowheads indicate shrunk and eosinophilic cytoplasm; scale bars represent 25 µm.

Photomicrographs of ovarian tissue were examined at every tenth (human) or sixth (mouse) section, and used for ovarian follicle counts and follicle stage and health assessment using ImageJ software by a blind-to-treatment assessor, as detailed in Morgan *et al*. (2013) (Fig. 1). In brief, follicles were staged as: primordial, when an oocyte with a visible germinal vesicle (GV) was surrounded only by flattened GCs (Fig. 1ai); transitional, when an oocyte with a visible GV was surrounded by a mixture of flattened and cuboidal GCs (Fig. 1aii); primary, when an oocyte with a visible GV was surrounded by a single layer of cuboidal GCs (Fig. 1aiii) or secondary, when an oocyte with a visible GV was surrounded by more than one layer of cuboidal GCs (Fig. 1aiv). Follicles were further classified as unhealthy (Fig. 1bi–iv) when containing: an oocyte with eosinophilic and shrunk cytoplasm, and/or condensed nuclear chromatin; GCs, the majority of which were irregularly shaped and/or had condensed chromatin; or those follicles with a combination of unhealthy oocytes and GCs.

For human ovary follicle assessment, where cultures had been carried out with small sections of an ovarian biopsy, follicle density per patient/treatment was then calculated by dividing the total number of follicles by the volume of tissue analysed and expressing this value as follicles per cubic millimetre. For each sample of human ovary, tissue volume was calculated as the sum of the area (mm^2^) of all tissue sections analysed multiplied by 0.5 µm (the thickness of each section), to give a value in mm^3^: this number was then multiplied by ten, the interval of sections counted, to get total density. Effects of drug exposure on follicle density were not examined where Control ovarian tissue had a density of less than 20 follicles per mm^3^, with five of the 14 ovarian tissue samples excluded from follicle analysis due to the low follicle density, giving a final sample size of nine for all human ovarian follicle analyses.

With the mouse ovary follicle assessment, it was possible to estimate total follicle numbers per ovary, since cultures had been carried out on whole ovaries; estimates of total follicle numbers were made by applying the Abercrombie correction to the counts of all visible GV in every sixth section (Abercrombie 1946).

### Immunohistochemistry

Slides of cultured human ovary were examined to identify sections that did not contain ovarian follicles, with reactions carried to determine rates of proliferation through IHC for BrdU incorporation (rat anti-BrdU antibody, Abcam, dilution 1:200) and of apoptosis through IHC for cleaved caspase 3 (CC3; rabbit anti-CC3 antibody, Cell Signalling Technology, dilution 1:500); *n* = 11 for both. Washes in PBS (Fisher Scientific UK Ltd) with 0.1% Triton X (PBSTx) were performed between each step. Antigen retrieval was performed in 0.01 M citrate buffer (pH 6, Sigma Aldrich Ltd), followed by blocking step with 20% normal goat serum diluted in PBSTx and 5% w/v BSA for 1 h at RT. Slides were incubated with primary antibodies overnight at 4°C in a humidified environment followed by incubation with appropriate secondary antibody and visualisation reagent all at 1:200 dilution: for BrdU IHC, AlexaFluor 568 nm goat anti-rat (Invitrogen) was used; for CC3 IHC, goat anti-rabbit biotinylated (Vector Laboratories), was followed by Streptavidin 488 (Vector Laboratories). Counterstaining was with 4,6-diamidino-2-phenylindole (DAPI; Invitrogen) at 1:5000 for 10 min and slides were then mounted with Vectashield hard-set mounting medium (Vector Laboratories).

### Image acquisition and analysis

Images were taken using a Leica DM5500B microscope with a DFC360FX camera. Image analysis was performed using ImageJ, with the assessor blind to treatment. For BrdU and CC3 IHC, fluorophore area was measured as a percentage of the section area, as in Lopes *et al*. (2016).

### Statistical analysis

Data were analysed using GraphPad Prism. One-way ANOVA (for normally distributed data) or the Kruskal–Wallis test (where data were not normally distributed) was performed to determine if there was an effect of treatment. Where these were significant, *post hoc* tests were carried out to determine statistical significance between both between control and different drug treatments and also between the combination treatment and treatment by a single drug (Single CIS, Double CIS, Single DOX or Double DOX), using Dunnet’s or Dunn’s tests respectively. All results are given as mean + s. e.m., with results considered statistically significant where significance was *P* < 0.05.

## Results

### Culture of human ovarian cortical slices supports follicle development

**Figure 2:**
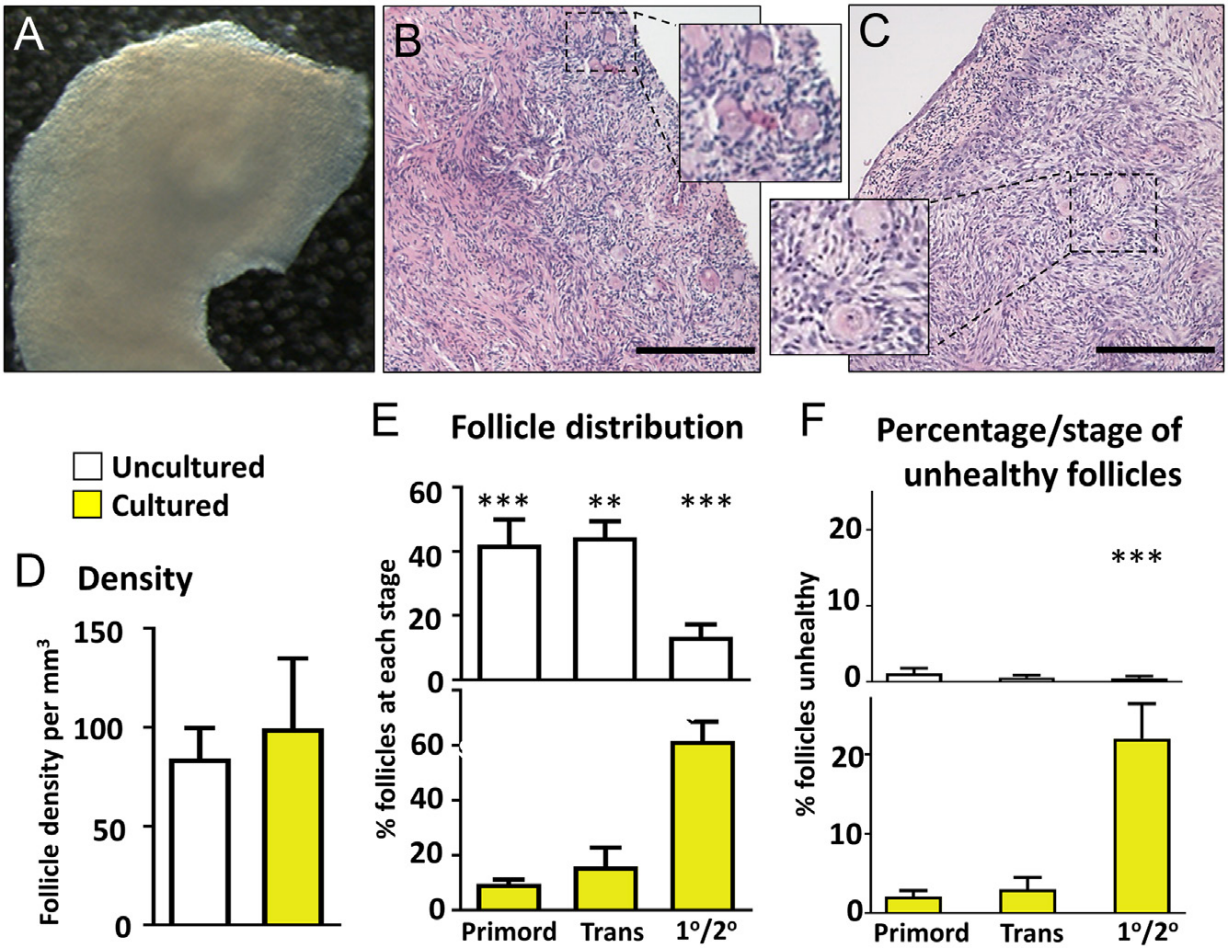
Effect of culture on human ovarian follicles. Culture of cortical strips from human ovary for 6 days supports follicles development. (A) Slice of ovarian cortex floating on a membrane in an air-liquid interface culture. (B and C) Representative photomicrographs of sections of ovarian tissue fixed in Bouin’s and stained with haematoxylin and eosin; insets are higher magnification of framed areas highlighting group of follicles: (B) – uncultured; (C) – cultured. (D, E and F): Histograms showing analysis of uncultured (white) and cultured (yellow) human ovarian tissue: (D) Follicle density (number per mm^3^); (E) Percentage of total follicles that were at primordial, transitional or primary/secondary stage of development; (F) Percentage of follicles that were unhealthy, at primordial, transitional and primary/secondary stage of development. Scale bars represent 200 µm. Histograms: data are mean + s.e.m.; asterisks denote significant differences between uncultured and cultured ovary – *P* < 0.01 (**), *P* < 0.001 (***). *n* = 9 for both uncultured and cultured groups. Primord – primordial follicles; Trans – transitional follicles; 1°/2° – primary/secondary follicles.

**Figure 3:**
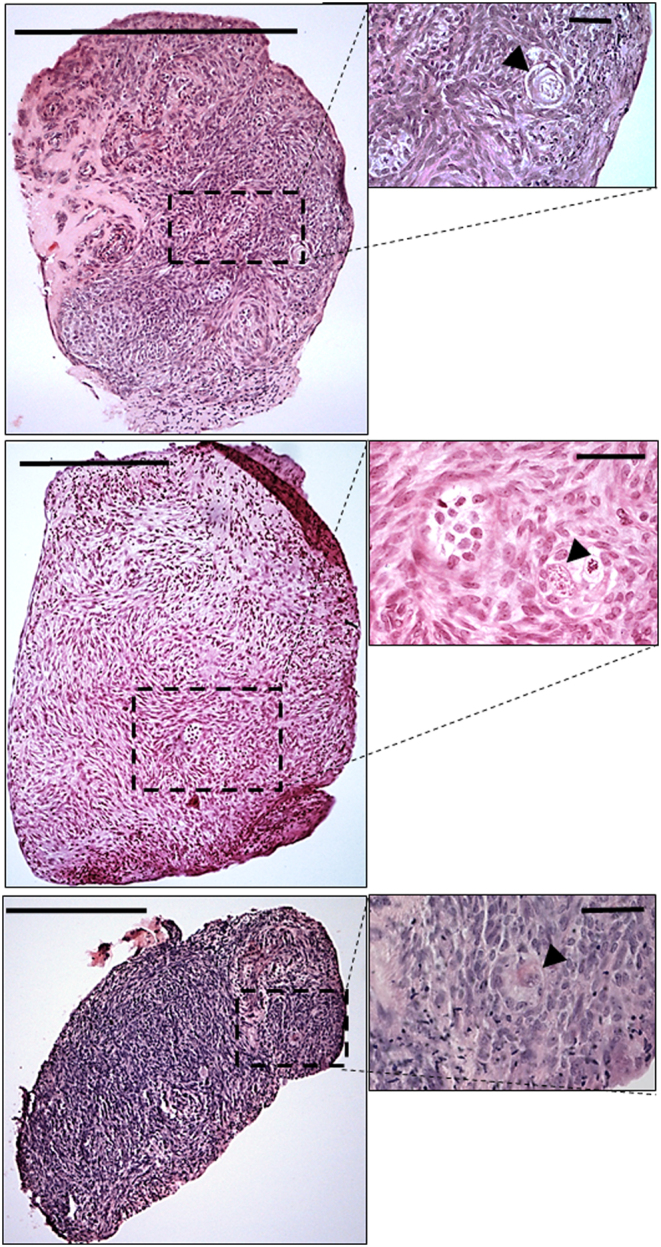
Examples of Control sections of cultured human ovarian fragments that contain unhealthy follicles. Photomicrographs of histological sections from three pieces of Control cultured ovaries, at low power (left), with unhealthy follicles shown in insets (right). Unhealthy follicles are randomly found throughout the sections, and not located in any one region. Black arrowheads indicate atretic follicles; scale bars represent 200 µm, or 25 µm for insets.

Overall, human ovarian strips cultured in an air-medium interface remained healthy across the culture period (Fig. 2B and C), containing follicles at the primordial through to the secondary stage. Analyses of follicle density and stage showed that the human ovary culture technique maintained follicle density (Fig. 2D), while supporting follicle development; uncultured ovary tissue contained primarily primordial and transitional follicles, while the cultured ovary contained mainly primary and secondary follicles (Fig. 2E). While follicle health was for the most part retained through the culture process (around 80% healthy follicles), there was an increase in unhealthy follicles, due to a significantly higher percentage of unhealthy primary and secondary follicles (*P* < 0.001; Fig. 2F). Examination of histological images showed that unhealthy growing follicles in the Control tissue were scattered across the whole section area, excluding the possibility that the necrosis of the centre of the tissue was responsible for the occurrence of unhealthy follicles (Fig. 3).

### Damage to ovarian follicles resulting from combined exposure to single doses of cisplatin and doxorubicin does not exceed that from exposure to each drug alone

Exposure to CIS and/or DOX was damaging to ovarian follicles in human and mouse ovary. With human ovary cultures, results of follicle counts are shown as follicular density since analysis is of a small fragment of ovary; in contrast, culture of whole mouse ovaries allows for analysis of total follicle numbers.

**Figure 4:**
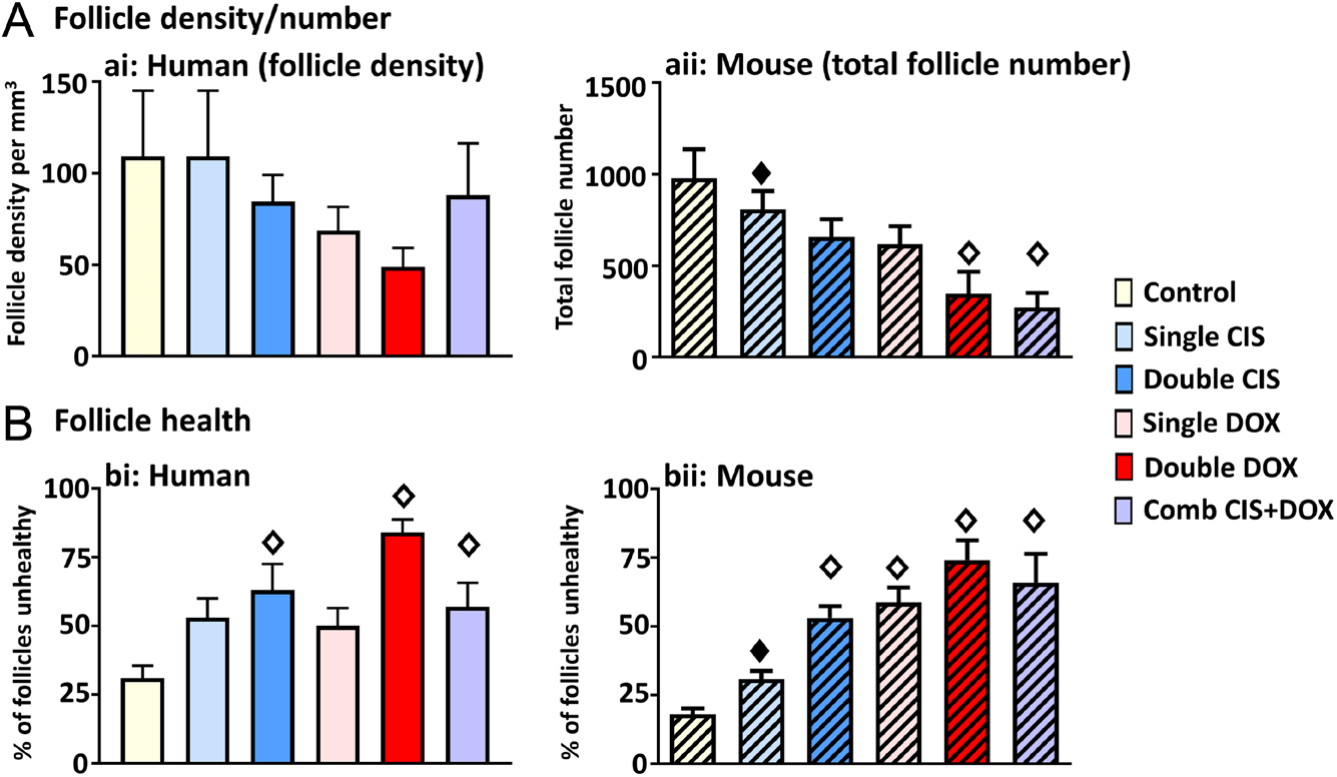
Effect of chemotherapy drug exposure on follicle density/number and health. Exposure to chemotherapy drugs affects follicle number (mouse) and health (human and mouse). (A and B): Follicle analysis in Control cultures, or after exposure to Single CIS, Double CIS, Single DOX, Double DOX or Combination CIS + DOX. (A) Density (ai: human – unhatched) or number (aii: mouse – hatched) of follicles. (B) Percentage of follicles that were unhealthy (bi: human – unhatched; bii: mouse – hatched). Data are mean + s.e.m.; unfilled diamonds denote significant differences between Control and a treatment group; filled diamonds denote significant differences between Combination CIS + DOX and another treatment group (individual *P* values are given in text). For human ovaries, *n* = 9 in each group; for mouse ovaries, 5–8 ovaries were analysed per treatment group as follows: Control – *n* = 7, Single CIS – *n* = 6, Single DOX – *n* = 6, Double CIS – *n* = 6, Double DOX – *n* = 5, CIS + DOX – *n* = 8). Comb – Combination.

Human ovary appeared to show a trend for drug exposure to result in lower follicle density, but there was no significant effect overall (Fig. 4ai; *P* = 0.4). Follicle health was affected by drug exposure (Fig. 4bi). There was no significant effect on follicle health from exposure to a single dose of either drug (Single CIS or Single DOX), but, compared to Controls, a significantly higher percentage of follicles were unhealthy after exposure to Double CIS (2.0-fold difference, *P* < 0.01), Double DOX (2.7-fold difference, *P* < 0.001) or to Combination CIS + DOX (1.8-fold difference, *P* < 0.05). There was no significant difference between the Combination CIS + DOX treatment and treatment by CIS or DOX alone, either Single or Double dose (Fig. 4ai and bi).

The mouse ovary showed a similar pattern of effect to the human ovary, although here there were statistically significant effects on follicle numbers (Fig. 4aii) and health (Fig. 4bii). Total follicle numbers were significantly decreased over those of Controls after exposure to Double DOX and to Combination CIS + DOX (Fig. 4aii: Double DOX – 2.8-fold difference, *P* < 0.01; Combination CIS + DOX – 3.6-fold difference, *P* < 0.001). In addition, the Combination CIS + DOX treatment was significantly different from the Single CIS treatment (Fig. 4aii: 3.0-fold difference, *P* < 0.05), but there was no significant difference between the Combination CIS + DOX treatment and any other treatment group. The percentage of follicles classified as unhealthy was increased over that of Controls in response to every treatment apart from Single CIS (Fig. 4bii: Double CIS – 3.0-fold difference, *P* < 0.01; Single DOX – 3.3-fold difference, *P* < 0.01; Double DOX – 4.1-fold difference, *P* < 0.001; and Combination CIS + DOX – 3.7-fold difference, *P* < 0.001). Notably though, the Combination CIS + DOX treatment did not lead to greater damage than Double CIS, Single or Double DOX, with Combination CIS + DOX treatment significantly different only to the Single CIS treatment (Fig. 4bii: 2.1-fold difference, *P* < 0.01).

### Density/number and health of follicles at specific stages following drug exposure

**Figure 5:**
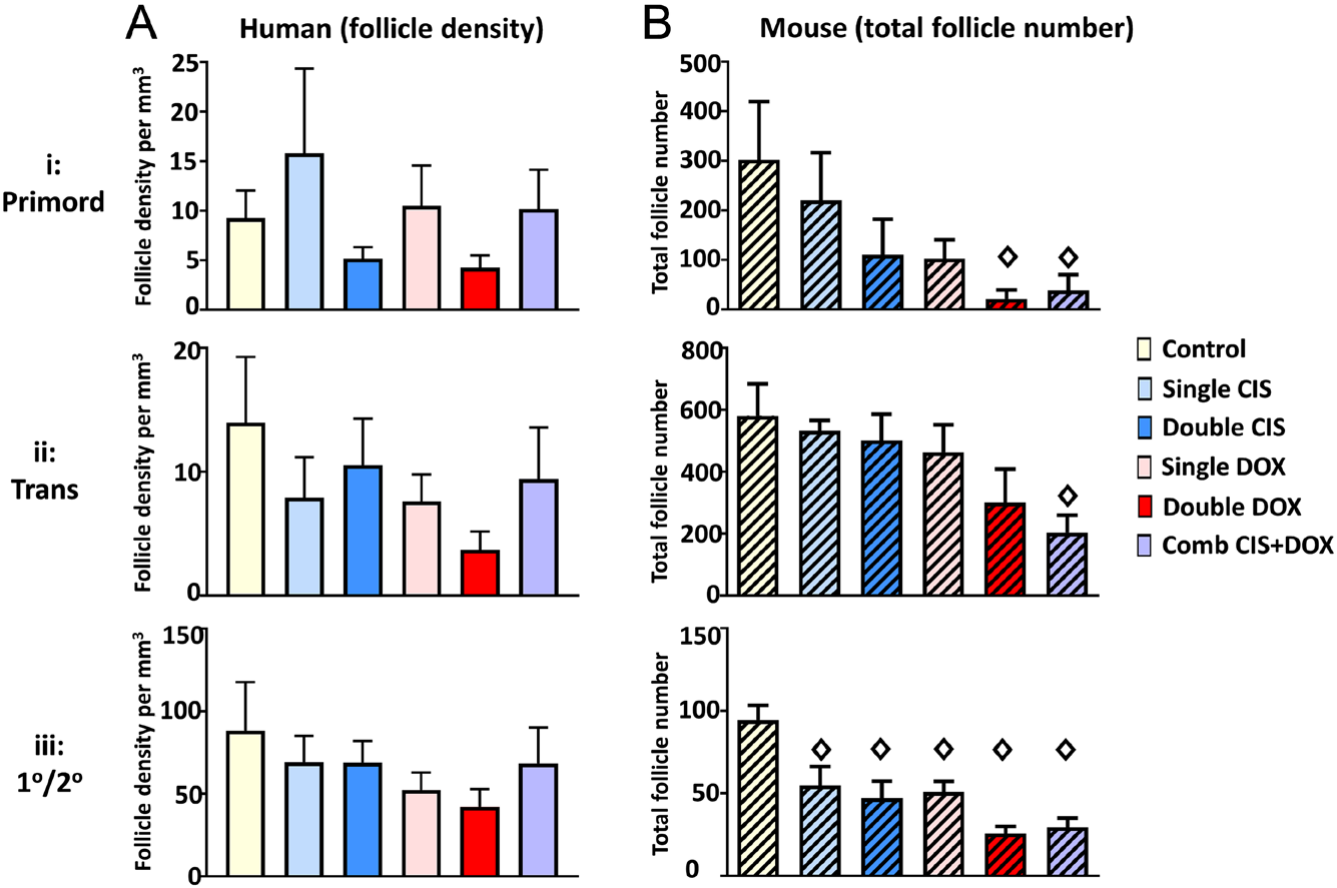
Effect of chemotherapy drug exposure on follicle density/number at each follicular stage. Follicle analysis in Control cultures, or after exposure to Single CIS, Double CIS, Single DOX, Double DOX or Combination CIS + DOX. (A) Density (human – unhatched) or (B) number (mouse – hatched) of follicles at (ai, bi) Primordial, (aii, bii) Transitional and (aiii, biii) Primary/Secondary stage. Data are mean + s.e.m.; unfilled diamonds denote significant differences between Control and a treatment group (individual *P* values are given in text). For human ovaries, *n* = 9 in each group; for mouse ovaries, 5–8 ovaries were analysed per treatment group as follows: Control – *n* = 7, Single CIS – *n* = 6, Single DOX – *n* = 6, Double CIS – *n* = 6, Double DOX – *n* = 5, CIS + DOX – *n* = 8). Primord – primordial follicles; Trans – transitional follicles; 1°/2° – primary/secondary follicles; Comb – Combination.

Follicles were classified as being at the primordial, transitional or primary/secondary stage, with analysis of numbers and the percentages that were healthy. There was no significant effect of drug on density of individual follicle stages in the human ovary at any individual follicle stage (Fig. 5ai–iii), although there was a trend for the density of primary/secondary follicles to be reduced following drug exposure (Fig. 5aiii; *P* = 0.32). Analysis of mouse ovaries showed effects of drug-exposure on follicles at all stages when compared with numbers in Control ovaries (Fig. 5B). Primordial and transitional follicle numbers (Fig. 5bi–ii respectively) were lower than Controls only after exposure to Double DOX (primordial follicles only: 14-fold difference, *P* < 0.05) or Combination CIS + DOX (primordial follicles – 8-fold difference, *P* < 0.05; transitional follicles – 2.6-fold difference, *P* < 0.05). In contrast, numbers of primary/secondary follicles were most clearly affected, being significantly lower than Controls after exposure to every drug treatment (Fig. 5biii: Single CIS – 1.7-fold difference, *P* < 0.05; Double CIS – 2.0-fold difference, *P* < 0.01; Single DOX – 1.9-fold difference, *P* < 0.01; Double DOX – 3.7-fold difference, *P* < 0.001; and Combination CIS + DOX – 3.2-fold difference, *P* < 0.001). No significant difference was found between Combination CIS + DOX and treatment by CIS or DOX alone, either Single or Double dose, for any ovarian follicle stage (Fig. 5bi–iii).

**Figure 6:**
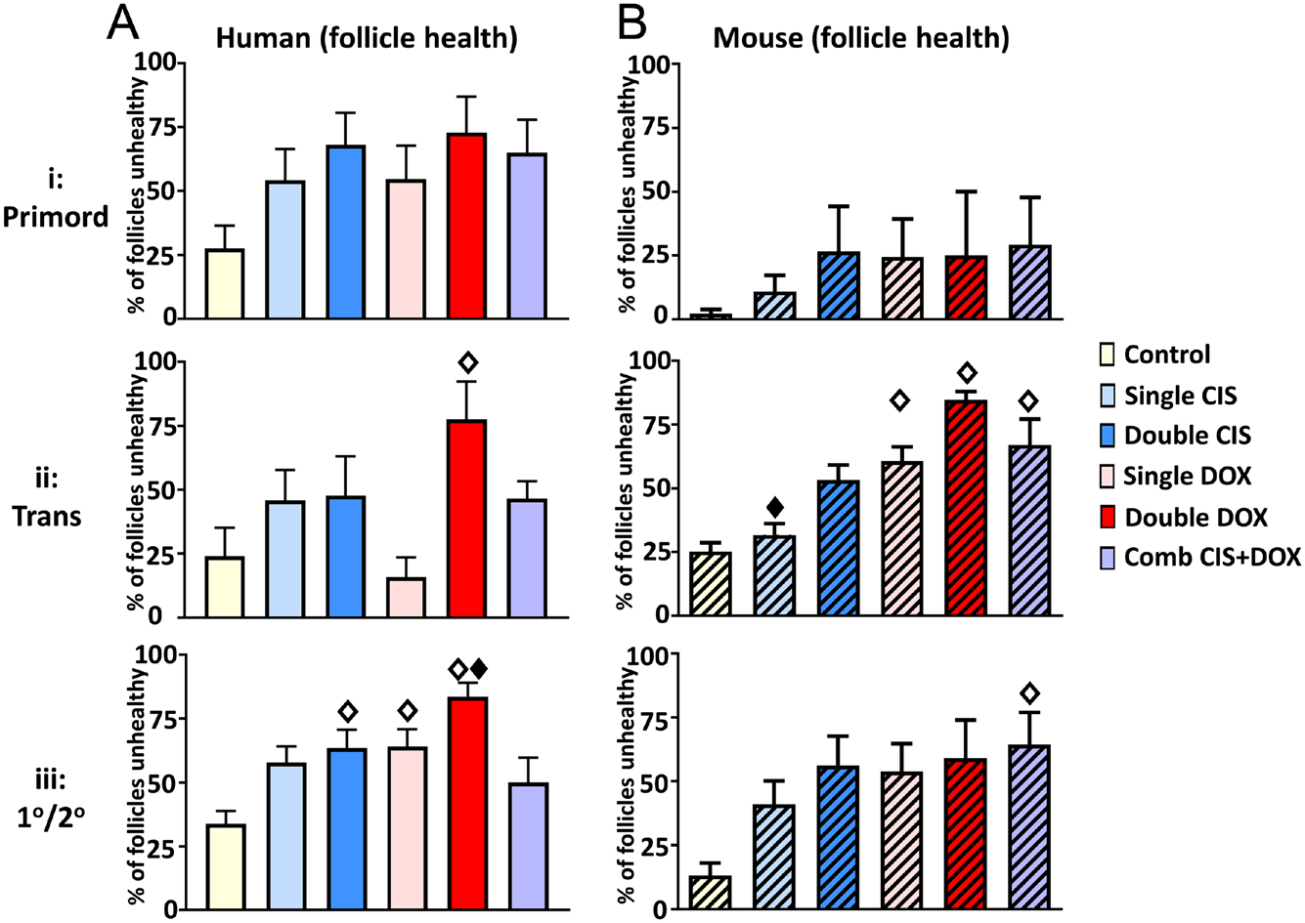
Effect of chemotherapy drug exposure on follicle health at each follicular stage. Follicle analysis in Control cultures, or after exposure to Single CIS, Double CIS, Single DOX, Double DOX or Combination CIS + DOX. (A and B) Percentage of follicles that are unhealthy; (A) human – unhatched; (B) mouse – hatched, at (ai, bi) Primordial, (aii, bii) Transitional and (aiii, biii) Primary/Secondary stage. Data are mean + s.e.m.; unfilled diamonds denote significant differences between Control and a treatment group; filled diamonds denote significant differences between Combination CIS + DOX and another treatment group (individual *P* values are given in text). For human ovaries, *n* = 9 in each group; for mouse ovaries, 5–8 ovaries were analysed per treatment group as follows: Control – *n* = 7, Single CIS – *n* = 6, Single DOX – *n* = 6, Double CIS – *n* = 6, Double DOX – *n* = 5, CIS + DOX – *n* = 8. Primord – primordial follicles; Trans – transitional follicles; 1°/2° – primary/secondary follicles; Comb – Combination.

There was a significant effect of drug exposure on the percentage of transitional and primary/secondary follicles that were healthy, in both human and mouse ovaries, while the trend for drug exposure to affect the health of primordial follicles was not significant (Fig. 6ai: human, *P* = 0.12; Fig. 6bi: mouse, *P* = 0.63). In the human cultured ovaries, transitional follicles exhibited a significant effect on health only after exposure to Double DOX (Fig. 6aii: Double DOX – 3.2 fold difference, *P* < 0.05), whereas in the primary/secondary follicles, there was an increase in the percentage of follicles that were unhealthy after exposure to Double CIS, Single DOX or Double DOX (Fig. 6aiii: Double CIS and Single DOX – 1.9-fold difference in both cases, *P* < 0.05; Double DOX – 2.5-fold increase, *P* < 0.001). The Combination CIS + DOX treatment was significantly different only to the Double DOX treatment, with significantly higher percentages of follicles unhealthy after exposure to Double Dox (Fig. 6aiii: 1.7-fold difference, *P* < 0.05). In the cultured mouse ovaries, when compared with Controls, transitional follicles exhibited a significant effect on health after exposure to Single DOX, Double DOX and Combination CIS + DOX (Fig. 6bii: Single DOX – 2.4-fold difference, *P* < 0.05; Double DOX – 3.4-fold difference, *P* < 0.001; Combination CIS + DOX – 2.7-fold difference, *P* < 0.01), whereas in the primary/secondary follicles, there was an increase in the percentage of follicles that were unhealthy only after exposure to the Combination CIS + DOX treatment (Fig. 6biii: Combination CIS + DOX – 4.9-fold increase, *P* < 0.05). The Combination CIS + DOX treatment was significantly different from other treatments only for transitional follicles in the Single CIS treatment group (Fig. 6biii: 2.2-fold difference, *P* < 0.05).

**Figure 7:**
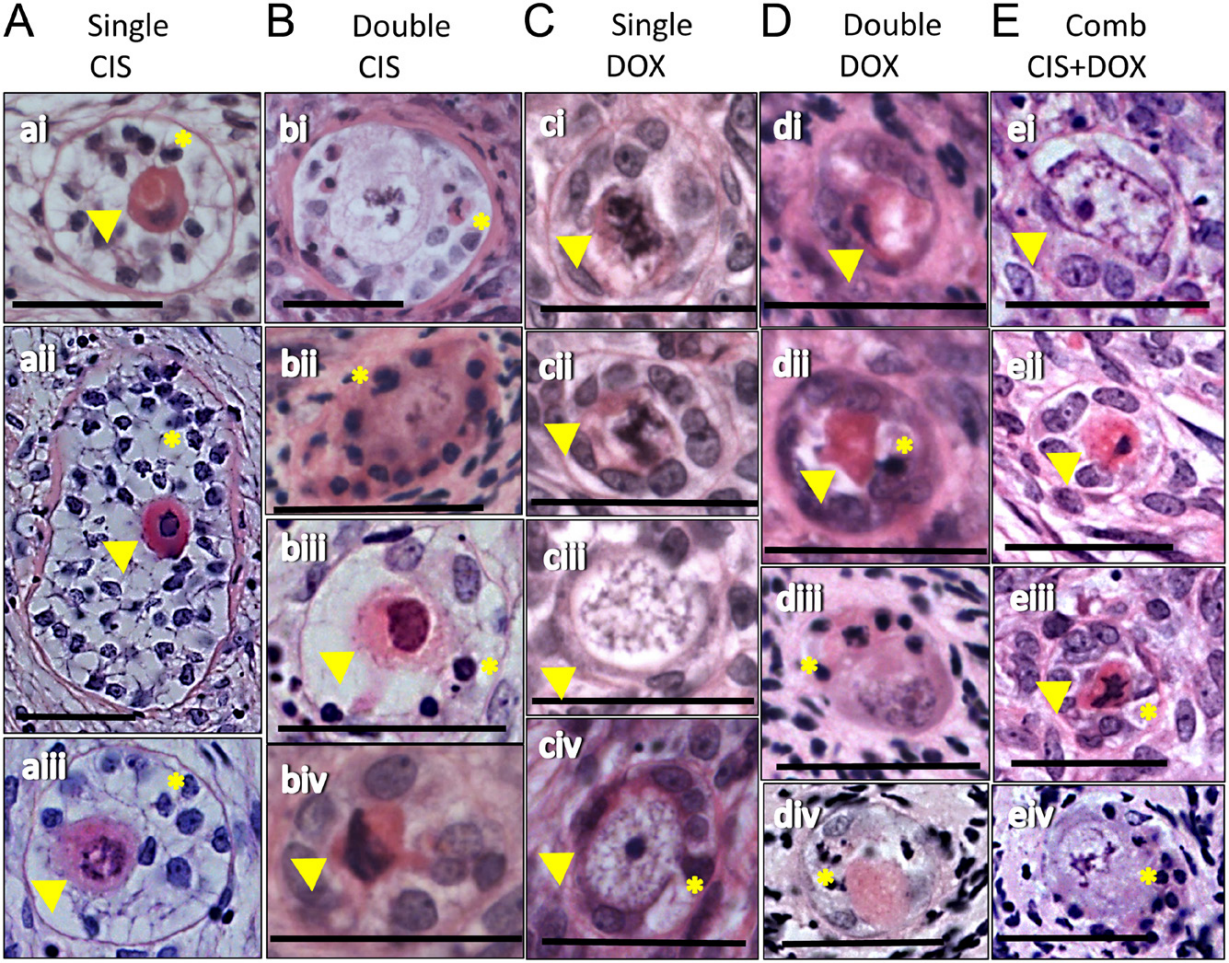
Histological images of unhealthy follicles exposed to chemotherapy drugs. Photomicrographs of histological sections of follicles from cultured ovaries that have been exposed to chemotherapy drugs. Follicles had been exposed to: (ai) Single CIS, (bi–iv) Double CIS, (ci–iv) Single DOX, (di–iv) Double DOX or (ei–iv) Combination CIS + DOX. Yellow arrowheads indicate shrunk and eosinophilic cytoplasm; yellow asterisks indicate condensed chromatin; scale bars represent 25 µm.

No specific pattern was observed in terms of cell type targeted by the drugs, with either oocyte and/or GCs showing signs of degenerations across treatments (Fig. 7). As with unhealthy follicles in Control sections (Fig. 3), unhealthy follicles in drug-exposed sections were found to be randomly distributed across the histological sections (see Supplementary Fig. 1, see section on supplementary materials given at the end of this article, for low power images of the drug-exposed sections that contained the unhealthy follicles shown in Fig. 3).

### Drug exposure leads to changes in apoptosis and proliferation in human ovarian stromal tissue

**Figure 8:**
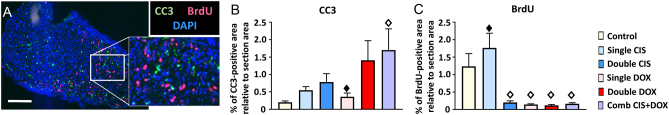
Apoptosis and cell proliferation in cultured human ovary exposed to chemotherapy drugs. Exposure of human ovary to chemotherapy drugs increases apoptosis and reduces cell proliferation. (A) Representative photomicrograph of cultured human ovary (inset is higher magnification of framed area), showing immunohistochemical localisation of cleaved caspase 3 (CC3) (green) and BrdU (red), counterstained with DAPI (blue). (B and C) Graphs show protein expression calculated as a percentage of area of ovary for (B) CC3 and (C) BrdU after exposure to Single CIS, Double CIS, Single DOX, Double DOX or Combination CIS + DOX. Scale bar represents 200 µm. Data are mean + s.e.m.; unfilled diamonds denote significant differences between Control and a treatment group; filled diamonds denote significant differences between Combination CIS + DOX and another treatment group (individual *P* values are given in text). Sample size: *n* = 11 in all groups. Comb – Combination.

Sections of cultured human ovarian stromal tissue that contained no ovarian follicles were examined for the effect of CIS and/or DOX on both apoptosis and cell proliferation, carrying out IHC for CC3 and BrdU respectively (Fig. 8A).

There was a trend for increased expression of CC3 after drug exposure, although that was significant to Controls only after exposure to the Combination CIS + DOX treatment (Fig. 8B: Combination CIS + DOX – 8.5-fold difference, *P* < 0.01). Amongst treatment groups, Combination CIS + DOX treatment was significantly different only from the Single DOX treatment (Fig. 8B: 4.7-fold difference, *P* < 0.05).

The strongest effect of drug exposure was found when investigating cell proliferation, despite the fact that the assessment was carried out by determining uptake of BrdU (incorporated into any cells entering the S-phase of the cell cycle) only during last day (Day 6) of culture, with no exposure of the tissue to chemotherapy drugs since Day 3. BrdU incorporation was significantly lower than that of Control ovaries after exposure to Double CIS, Single DOX, Double Dox and Combination CIS + DOX (Fig. 8C: Double CIS – 6.2-fold difference, *P* < 0.05; Single DOX – 8.8-fold difference, *P* < 0.01; Double DOX – 10.2-fold difference, *P* < 0.001; Combination CIS + DOX – 7.7-fold difference, *P* < 0.01). Amongst treatment groups, Combination CIS + DOX treatment was significantly different only to the Single CIS treatment (Fig. 8C: 4.7-fold difference, *P* < 0.05).

## Discussion

It has long been recognised that chemotherapy treatment can result in reduced fertility for females. However, clinical information predominantly comes from patients who are administered complex combinations of drugs, while the literature mainly examines direct effects of the drugs on the ovary, using the mouse as a model, and with a lack of investigations into potential combinatory effects of chemotherapy drugs. Here, we show the effects of exposing the human ovary to CIS and/or DOX, through use of a tissue culture system that we show is able to support the development and growth of early stage human ovarian follicles. The work investigated the impact of the chemotherapy drugs on both the follicular and stromal ovarian compartments, and examined the combined effects of CIS and DOX by comparing the effect of exposure to single or double doses of CIS or DOX alone with exposure to a combination treatment of a single dose of CIS + DOX. While exposure to either CIS or DOX alone reduced ovarian health, there was no evidence of any multiplicative effects when ovaries were cultured in a combined CIS + DOX treatment. Effects of treatment had a similar pattern in the mouse ovary, both qualitatively and quantitatively; however, here differences more often reached statistical significance, supporting the value of the mouse as a model for the human ovary for toxicological studies. Given the importance of the stromal compartment of the ovary, and the relatively high proportion of the human ovary that is composed of stromal tissue, the effect of the drugs on human ovarian cortical stromal tissue was evaluated: drug treatment resulted in increased apoptosis, and in a striking reduction in cell proliferation.

CIS is a platinum-based alkylating-like agent, usually considered to have moderate-to-high gonadotoxicity. It acts by inducing DNA cross-linking that results in the formation of adducts which interfere with DNA repair and block cell division: together this leads to DNA damage, inducing apoptosis (Dasari & Tchounwou 2014). It has been suggested that CIS also interferes with nuclear and cellular proteins connected with its long-term toxic effects (Chovanec *et al*. 2017). DOX is an anthracycline and an inhibitor of topoisomerase II (Topo II); clinically, the range of reported gonadotoxicity for DOX is wide (Ben-Aharon *et al*. 2010, Soleimani *et al*. 2011). DOX inhibits Topo II by inhibiting resealing cleaved DNA, thus leading to increased DNA fragmentation and hence cell death (Kiyomiya *et al*. 1998, Thorn *et al*. 2011). DOX also acts on mitochondria, reducing both mitochondrial membrane potential and cytochrome C release.

Modern chemotherapy treatment involves the combined administration of multiple drugs. Combination therapy has proved key to improving treatment efficiency, likely due to the different drugs targeting differing pathways, thus resulting in additive or even synergistic effects (Bayat Mokhtari *et al*. 2017). For CIS and DOX in particular, the addition of DOX has proved to increase significantly CIS cytotoxicity on cancer cells, with both clinical studies on cancer patients (Bruckner *et al*. 1981) and also* in vitro* studies using cancer cell lines (Xu *et al*. 1989) showing that CIS and DOX can have additive effects: hence, their common combined use. However, less is known about any potential undesired consequences of such a multi drug regimen, in particular on female fertility, and it is important to bear in mind that any off-target effects resulting from combined treatment could be more severe (Calabrese 1995, Delbaldo *et al*. 2004). Here, no multiplicative effects on the ovary were found: in no case did exposure to a combined single dose of CIS and DOX together result in more severe damage than that found after exposure to a double dose of either CIS or DOX alone.

Treatment with either CIS or DOX resulted in a reduction in follicle number and increase in the percentage of unhealthy follicles in human and mouse ovary, with significant effects on the number and health of the total follicle population. This was due to an effect on the population of growing follicles in particular. These results are consistent with previous work, and add to the limited number of publications investigating effects of CIS or DOX on the human ovary. In the human ovary, CIS has been shown to affect follicle number in cultured ovarian cortical strips and in xenotransplantations (Bildik *et al*. 2015, 2018) and results in increased apoptosis in cultured luteinised GCs (Chatterjee *et al*. 2014, Yuksel *et al*. 2015, Bildik *et al*. 2018), while DOX leads to a reduction in follicle numbers and increase in the number of atretic follicles in xenotranplanted human ovarian cortical tissue (Soleimani *et al*. 2011, Li *et al*. 2014). The majority of our information about the effects of CIS or DOX on the ovary comes from studies using the mouse as a model. CIS results in oocyte damage (Kim *et al*. 2013, Nguyen *et al*. 2019), with oocytes activating c-ABL and in turn the oocyte-specific homologue of p53, Tap63, that mediates the oocyte’s DNA damage response (Gonfloni *et al*. 2009, Gonfloni 2010, Bolcun-Filas *et al*. 2014, Rinaldi *et al*. 2017, Nguyen *et al*. 2018, Kim *et al*. 2019). CIS also increases phosphorylation of components of the PTEN/Akt/FOXO3a pathway that regulates growth activation of primordial follicles (Chang *et al*. 2015, Jang *et al*. 2016). DOX induces DNA damage in somatic cells (Roti Roti *et al*. 2012, 2014, Xiao *et al*. 2017).

Apoptosis and proliferation of ovarian cortical stromal tissue was affected by drug exposure. As with the follicular compartment, there was no evidence of any multiplicative effect between the two drugs, although only the co-treatment of CIS and DOX induced a significant increase in apoptosis in the stromal tissue compared to that of Controls. The effect on stromal tissue was most pronounced when examining cell proliferation, with a dramatic reduction in proliferating cells after exposure to all drug treatments other than Single CIS treatment. Notably, this was despite the fact that exposure to the drugs had ended 96 h prior to tissue examination, with all tissue maintained in drug-free medium throughout that 96-h period. Effects on apoptosis in human ovarian cells have been described previously, with Fabbri *et al*. (2016) showing effects of both CIS and DOX on cultured primary stromal cells, and Bildik *et al*. (2018) demonstrating increased apoptosis in cultured luteinised granulosa cells. Both CIS and DOX have also been shown to damage the stromal vasculature (Meirow *et al*. 2007, Soleimani *et al*. 2011, Bildik *et al*. 2015). To the best of the authors’ knowledge, this is the first report on effects of CIS or DOX on human stromal cell proliferation, with the effects of chemotherapy drugs on the stromal component of the ovary examined relatively little to date (Spears *et al*. 2019). A decrease in stromal cell proliferation could result in reduced formation of thecal cell layers in developing follicles, or of neoangiogenesis, either of which would compromise follicle growth and development.

The effects of CIS and/or DOX on ovarian follicles was examined both in the human and also the mouse ovary in order to assess how effective the mouse ovary is as a model of the human ovary. The same experimental paradigm was used in each case, exposing ovaries to single or double doses of each drug alone, or to a combination treatment of a single dose of both CIS and DOX together. Lower drug concentrations were required for the mouse ovary compared to the human ovary to induce similar levels of damage, most likely due to the lower density of stromal compartment in mouse ovary. CIS was administered at 5 or 10 μg/mL (equivalent to 16.7 or 33.4 μM) in the human ovary cultures, and to 0.5 or 1.0 μg/mL in the mouse; DOX exposure was to 1 or 2 μg/ml (equivalent to 1.7 or 3.4 μM) in the human ovary cultures, and to 0.05 or 0.1 μg/mL in the mouse. This compares to concentrations of 20–40 μg/mL of CIS (Bildik *et al*. 2015, 2018) and 1, 10 or 100 μg/mL of DOX (Soleimani *et al*. 2011) previously used in human ovarian cortical tissue culture. Overall, the same pattern of effect was found in the human and mouse ovary, with statistically significant effects more frequent in the mouse ovary study than in the human. The increased statistical power of the mouse study is most likely due to the use of an inbred strain of mouse, compared to the challenging variability in the samples of human ovarian tissue. In addition, cultures of neonatal mouse ovary can use intact, whole ovaries, enabling estimations of total follicle numbers, whereas samples of human ovary require analysis of follicle densities, despite the high variability in follicle density across the human ovary. Taken together, this points to the suitability of using the mouse as a model in studies such as these.

In summary, this study examines the individual and combined effects of two commonly co-administered chemotherapy drugs, cisplatin and doxorubicin, using both human and mouse ovarian tissue. Cisplatin and doxorubicin both impacted on the health of ovarian follicles and stromal tissue, with results in the mouse study closely mirroring those using human tissue, giving weight to use of the mouse as a model species in these kinds of toxicological studies. Importantly, no evidence was found of any multiplicative effects between the two drugs.

## Supplementary Material

Supplementary Figure 1. Representative images of human ovarian cortex treated with chemotherapy drugs. Low magnification images of tissue from which follicles shown in Figure 7 were obtained.

## Declaration of interest

Norah Spears is on the editorial board of *Reproduction*. Norah Spears was not involved in the review or editorial process for this paper, on which she is listed as an author. The other authors have nothing to disclose.

## Funding

Work supported by Medical Research Grant (MRC) grant G1002118. J L was supported by 2014 Fujian Province Scholarship funding.

## Author contribution statement

F L, J L participated in the experimental design of the study, led experiments, analysed data, prepared figures and helped draft the manuscript; S M participated in the experimental design of the study and in experiments; R M, L N helped carry out experiments and analyse data; R A A conceived and designed the study, analysed data and helped draft the manuscript; N S conceived, designed and coordinated the study and wrote the manuscript. All authors read and approved the final version of the manuscript.
