# Supplementary material for: Single and combined effects of cisplatin and doxorubicin on the human and mouse ovary in vitro
Source: Reproduction. 2019 Dec 9;159(2):193–204. doi: 10.1530/REP-19-0279 (PMC6993208; doi:10.1530/REP-19-0279)

Single and combined effects of cisplatin and doxorubicin on the human ovary in vitro.

Lopes, Liu, Morgan, Matthews, Nevin, Anderson and Spears.

Supplementary Figure 1. Representative images of human ovarian cortex treated with chemotherapy drugs. Low magnification images of tissue from which follicles shown in Figure 7 were obtained.

Lettering within each image shows relevant panel(s) of Figure 7.

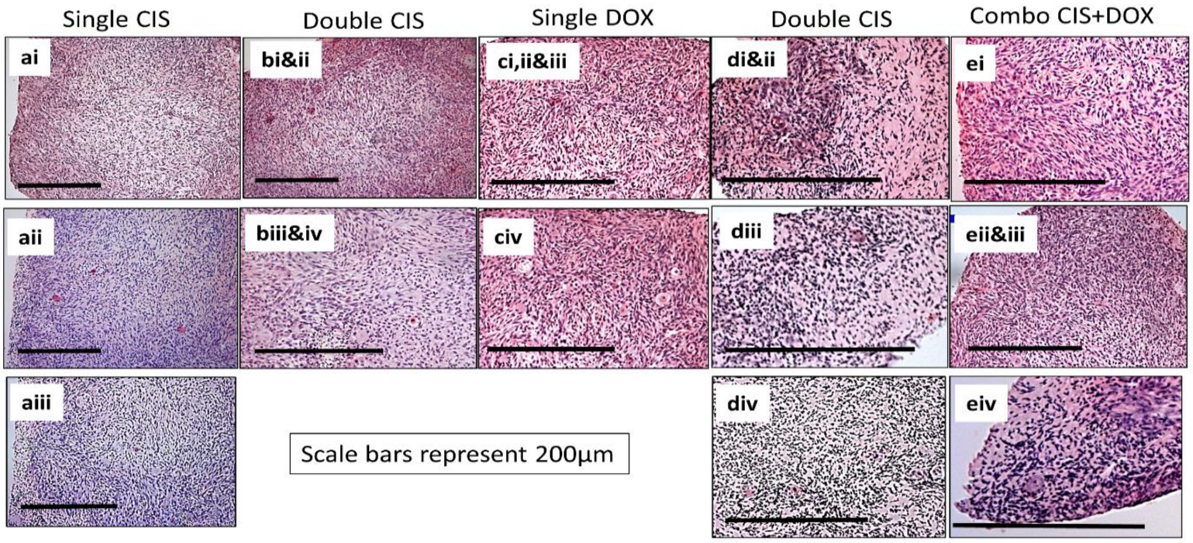

Supplement: Supplementary Figure 1. Representative images of human ovarian cortex treated with chemotherapy drugs. Low magnification images of tissue from which follicles shown in Figure 7 were obtained. [file supplementary_figure_1.pdf]
